# Antidepressant Effect of *Heracleum moellendorffii* Extract on Behavioral Changes in Astrocyte Ablation Mouse Model of Depression by Modulating Neuroinflammation through the Inhibition of Lipocalin-2

**DOI:** 10.3390/nu16132049

**Published:** 2024-06-27

**Authors:** Soonsang Hong, Yunna Kim, YongJu Kwon, Seung-Hun Cho

**Affiliations:** 1 Department of Clinical Korean Medicine, Graduate School, Kyung Hee University, Seoul 02447, Republic of Korea; soon0506@gmail.com (S.H.); naengcoffee@daum.net (Y.K.); 2 Department of Neuropsychiatry, College of Korean Medicine, Kyung Hee University Medical Center, Kyung Hee University, Seoul 02447, Republic of Korea; yunna.anna.kim@khu.ac.kr; 3 Research Group of Neuroscience, East-West Medical Research Institute, WHO Collaborating Center, Kyung Hee University, Seoul 02447, Republic of Korea

**Keywords:** depression, *Heracleum moellendorffii*, L-α-aminoadipic acid, astrocyte, lipocalin-2, neuroinflammation

## Abstract

Astrocyte dysfunction and inflammation play a pivotal role in depression. In this study, we evaluated the antidepressant properties of *Heracleum moellendorffii* root extract (HME), which is traditionally used for inflammation-related diseases, in a mouse model with astrocyte depletion that resembles the prefrontal cortex pathology of depressive patients. Mice were divided into four groups, with 10 mice per group. To induce astrocyte ablation in the mice’s prefrontal cortex (PFC), we used astrocytic toxin L-alpha-aminoadipic acid (L-AAA) and administered HME orally at 200 and 500 mg/kg for 22 days. We utilized the tail suspension test (TST) to assess depression-like behaviors and the open field test (OFT) to evaluate anxiety-like activities. Additionally, astrocytic and inflammatory markers in the PFC were evaluated using immunohistochemistry and ELISA. The results showed that infusion of L-AAA significantly decreased the expression of astrocytic glial fibrillary acidic protein (GFAP), which was accompanied by increased depression and anxiety-like behaviors. However, HME significantly reversed these effects by dose-dependently enhancing GFAP expression and modulating inflammatory markers, such as TNF-α, IL-6, and particularly lipocalin-2, a master proinflammatory mediator. These results imply that HME contributes to the alleviation of depression and anxiety-like behaviors by promoting astrocyte recovery and reducing neuroinflammation, especially through lipocalin-2 inhibition.

## 1. Introduction

Depression is a common neuropsychiatric disorder that affects approximately 350 million people worldwide. Symptoms generally include anxiety, mood fluctuations, insomnia, anorexia, chronic fatigue, or suicidal tendencies [1,2]. It is a costly and multibillion-dollar annual major economic burden. Despite extensive therapeutic studies on depression, commonly used antidepressants have limitations, such as limited remission rates and side effects occurring in approximately 30% of patients [3,4]. According to the Sequenced Treatment Alternatives to Relieve Depression (STAR*D), the remission rate at the initial treatment level using selective serotonin reuptake inhibitors (SSRIs) was lower than 40%, and the cumulative remission rate over the course of four treatment levels was 67% [4].

A decrease in the number or function of astrocytes in the prefrontal cortex (PFC) is a hallmark of depression. Previous studies have shown that both in patients with depression and animal models of depression, the brain shows reduced numbers of astrocytes and increased levels of glutamate in the PFC [5]. Astrocytes, the most abundant glial cells, play a crucial role in the neuronal activity of extracellular environment maintenance by surrounding synapses and blood vessels [6,7]. As astroglial–neuronal interactions are important in brain transaction function and pathophysiological regulation, changes in astrocytes can cause subsequent abnormal conditions in patients with depression. According to recent studies, symptoms of depression can be induced in animal models by injecting L-alpha-aminoadipic acid (L-AAA), an astrocytic toxin, to cause astrocyte dysfunction [3,8]. This model is derived from previous studies in which autopsy of the brains of patients with depression demonstrated a reduction in the density and quantity of glial cells in the cortices, particularly in the prefrontal regions [9].

*Heracleum moellendorffii* extract (HME) is an edible wild herb belonging to the Umbelliferae family. It is mainly distributed in Korea, China, and Japan [10]. HME comprises a number of flavonoids, such as skimmin, monoterpenoids, sesquiterpenoids, isopimpinellin, and polyacetylenes [10]. HME is also reported to be effective for skin disease, diabetes, fever, pain, and arthritis and has pharmacological features, including sedative, anti-inflammatory, anti-oxidative, and detoxification effects [10,11,12]. Its root is used in traditional Korean medicine because of its outstanding effects on neuralgia and anti-inflammatory activity [10,13].

Despite its pharmacological effects and relevant usage, no previous studies have reported its potential antidepressant efficacy. Considering its effects on inflammation and neurological diseases, we investigated whether HME has the potential to exert antidepressant-like effects. This study investigated the effect of HME on the behavior of an L-AAA-induced depression model in mice and its underlying anti-inflammatory mechanism.

## 2. Materials and Methods

### 2.1. Plant Material Preparation

HME was harvested in May 2016 in Yeongwol, Republic of Korea. A dried voucher specimen was stored in the herbarium of the Department of Korean Neuropsychiatry at Kyung Hee Medical Center (register number KH-019). The dried HME roots were boiled in 30% ethanol for an hour, twice. The extract was then filtered with filter paper. A rotary evaporator was used to concentrate the extract. Afterward, a brown powder was produced by lyophilizing the decotion. About 8% (*w*/*w*) of the initial natural product was obtained from the dried extract. The extract was stored at 4 °C in a refrigerator in a sealed container with parafilm to maintain stability by minimizing microbial growth and chemical reactions. The experiment was performed immediately after lyophilization to ensure maximum stability and integrity of the extract.

The marker compounds, skimmin and isopimpinellin, were identified in the HME to confirm their quality. Identification of skimmin and isopimpinallin was performed via chromatographic analysis using ultra-performance liquid chromatography triple time-of-flight mass spectrometry/mass spectrometry (UPLC-Triple TOF-MS/MS). The analysis was performed using a Thermo Scientific Vanquish UHPLC system (Thermo Fisher Scientific, Sunnyvale, CA, USA) and a Triple TOF 5600+ mass spectrometer system (Triple TOF MS; QTOF, Sciex, Foster City, CA, USA). Formic acid (FA) was purchased from Sigma Aldrich (St. Louis, MO, USA) and used as the mobile phase. Water, ethanol, and acetonitrile (ACN) were purchased from Honeywell Burdick & Jackson (Muskegon, MI, USA). The reference standards, skimmin (CAS 93-39-0, RS877545) and isopimpinellin (CAS 482-27-9, RS888768), were purchased from Interpharm Inc. (Goyang, Republic of Korea). Stock solutions of the reference standards (1 μg, respectively) were dissolved in 1 mL of methanol. The dried HME powder was dissolved in methanol (50 mg/mL). A syringe filter (0.22 µm pore size) was used to filter the solution. An Acclaim RSLC 120 C18 (2.1 × 100 mm) column was used for the separation process. The settings were configured as follows: temperature set at 40 °C, flow rate at 0.5 mL/min, injection volume of 2 µL, and UV detector wavelength at 265 nm. The mobile phase comprised 0.1% FA in water (solvent A) and 0.1% FA in ACN (solvent B), with a gradient elution program.

### 2.2. Animals

Seven-week-old male C57Bl/6 mice (Orient Bio Inc., Seongnam, Republic of Korea), which weigh 20–22 g, were used in this experiment. The mice were housed under standardized conditions, maintaining a constant temperature of 22 ± 2 °C and relative humidity of 60 ± 10% in acrylic cages measuring 20 × 27 × 12 cm. A controlled 12-h light–dark cycle was established by illuminating the cages from 6:00 a.m. to 6:00 p.m. The mice were unrestrictedly provided with food and water. They were allowed to adapt for one week. Every behavioral test was carried out from 10:00 to 17:00. All experimental procedures were conducted in compliance with the Guide for the Care and Use of Laboratory Animals and Korean Animal Protection Act and in accordance with the regulations of the Kyung Hee University Medical Center Institutional Animal Care and Use Committee (approval number; KHMC-IACUC 16-033).

The mice were randomly divided into four groups: (i) Sham: sham surgery + vehicle administration (n = 10); (ii) L-AAA+Veh: L-AAA infusion + vehicle administration (n = 10); (iii) L-AAA+HME 200 mg/kg: L-AAA infusion + HME 200 mg/kg administration (n = 10); (iv) L-AAA+HME 500 mg/kg: L-AAA infusion + HME 500 mg/kg administration (n = 10).

### 2.3. Cannula Manufacturing and Implantation

We created a cannula to inject L-AAA bilaterally into a specific location to establish a mouse model of depression. The procedure was performed under a modified version of a previously published protocol [14,15]. The double cannula was elaboratively manufactured and consisted of a guide cannula, internal cannula, and injection cannula (Supplementary Figure S1A). All cannulas were fabricated using needles produced by BD Precision Glide ^TM^ needles (Becton Dickinson, Franklin Lakes, NJ, USA). The cannula was designed to be implanted for injection into the PFC region with the following stereotaxic coordinates: 1.7 mm anterior/posterior, ±0.3 mm medial/lateral, and −2.5 mm dorsal/ventral from the bregma (Supplementary Figure S1B) [16]. The guide cannula was made with a 24 G needle, and the internal and injection cannulas were made with a 30 G needle [17].

Mice underwent cannula implantation under ketamine/xylazine (100/10 mg/kg) anesthesia via intraperitoneal injection. Mice were placed on a stereotaxic apparatus (Vernier Stereotaxic Instrument, Leica Biosystems, Nussloch, Germany), and guide cannulas were implanted in the PFC region. All animals were allowed a 7-day recovery period.

### 2.4. L-AAA Injection and Drug Administration

After implantation, the mice were given a week to recover. Subsequently, L-AAA (100 μg/μL; Sigma Aldrich, St. Louis, MO, USA) and the vehicle were administered via the internal cannula using an injection cannula and a microdrive pump (Pump 11 Elite Nanomite, Harvard Apparatus, Holliston, MA, USA). L-AAA was infused once daily for 2 days at 0.1 μL/min for 6 min per side (1.2 μL/mouse). Sham mice underwent the same schedule without injection, while L-AAA+Veh, L-AAA+HME 200 mg/kg, and L-AAA+HME 500 mg/kg were injected with L-AAA.

**Figure 1:**
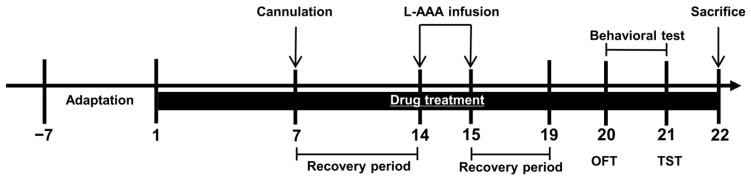
Experiment schedule. The timeline depicts the injection of L-AAA into the prefrontal cortex, as well as the drug treatment and behavioral tests. L-AAA, L-alpha-aminoadipic acid; OFT, open field test; TST, tail suspension test.

After the complete dissolution of HME in distilled water, the mice were orally administered doses of 200 mg/kg and 500 mg/kg of the HME solution. A vehicle of 0.2 mL distilled water was used for administration. Oral administration of the drug continued for 22 days until the animals were sacrificed. All the experiments followed the timetable shown in Figure 1.

### 2.5. Behavioral Tests

The open field test (OFT) is the most widely used test for assessing locomotor activity and anxiety. We performed the OFT as described previously [18,19]. Briefly, every mouse was put in the center of an arena made of opaque white acrylic boxes (50 × 50 × 50 cm), and its behavior was recorded for 10 min. The arena was subdivided into 25 equal squares: a ‘peripheral area’ consisting of the outer 16 squares and a ‘central area’ consisting of the inner nine squares. The total distance traveled by the mice was used to measure their horizontal locomotor activity. The center area travel distance of the mice was used to measure anxiety-like behavior. The distance was automatically assessed using a computer-aided control system (SMART, Panlab Harvard Apparatus, Holliston, MA, USA).

The mice’s locomotor activity and anxiety were assessed using the OFT. Depression-like behavior was estimated using the tail suspension test (TST). Briefly, adhesive tape was used to fix each mouse’s tail approximately 1 cm from the tip, and the mouse was suspended at a height of 50 cm from the floor. The test lasted for 6 min, during which the immobility time was determined by analyzing video recordings of the mice’s behavior during the final 4 min. All experiments were recorded on video.

### 2.6. Tissue Collection

To obtain brain tissue for immunohistochemical analysis, anesthesia and transcardiac perfusion were performed. Subsequent to anesthetization, the mice underwent perfusion with phosphate-buffered saline (PBS) followed by 4% paraformaldehyde. Following perfusion, the brains were carefully extracted and immersed in 4% paraformaldehyde for fixation at 4 °C for over 12 h. Upon completion of fixation, the brains were transferred to a 20% sucrose solution and stored at −80 °C until further processing for immunohistochemical analysis. For tissue collection for ELISA, mice were euthanized via cervical dislocation. Prefrontal cortices (PFC) dissected from the remaining mice were also preserved at −80 °C for subsequent enzyme-linked immunosorbent assay (ELISA).

### 2.7. Immunohistochemical Analysis

All brain samples were rapidly frozen and sectioned into 10 μm slices using a cryostat microtome (Leica CM1850; Leica Microsystems, Germany). Hematoxylin and eosin (H&E) staining was conducted to verify that cannula insertion had not induced any tissue damage in the prefrontal cortex (PFC).

Immunohistochemical studies were conducted. The following mouse monoclonal antibodies were used: anti-NeuN (MAB377) from Millipore Inc. (Billerica, MA, USA) and anti-β-actin (SC-69879) and anti-glial fibrillary acidic protein (GFAP, SC-33673) from Santa Cruz Biotechnology Inc. (Santa Cruz, CA, USA). The tissue sections were washed twice for 15 min in PBS. Subsequently, after blocking, they were incubated for 1 h in a solution containing 5% normal goat serum in PBS. The sections were incubated overnight at 4 °C with primary antibodies against NeuN (dilution 1:200) and GFAP (dilution 1:200). The sections were washed in PBS and treated with a biotinylated secondary antibody for 1 h at room temperature. The sections were subsequently incubated with an avidin-conjugated peroxidase complex (Standard Vectastain ABC Elite Kit, Vector Laboratories, Burlingame, CA, USA) for 1 h at room temperature for 1 h at room temperature. The sections were then washed with PBS, incubated in 3,3′-diaminobenzidine tetrahydrochloride (Dako, Carpenteria, CA, USA) as the chromogen, and washed with PBS. The sections were subjected to a dehydration procedure, which comprised washing with distilled water and dehydration in graded ethanol (70%, 95%, and 100%) and xylene. The sections were mounted using a permanent mounting medium on silane-coated slides (Muto Pure Chemicals Ltd., Tokyo, Japan), and microscopic analysis was performed using the Olympus BX51 microscope (Olympus, Tokyo, Japan).

### 2.8. Enzyme-Linked Immunosorbent Assay (ELISA)

PFC tissue was used for ELISA to determine tumor necrosis factor-α (TNF-α), interleukin 6 (IL-6), and lipocalin-2 (LCN2). Tissue lysates were quantified using an ELISA kit (TNF-α, BMS607-3, Invitrogen, Carlsbad, CA, USA; IL-6, BMS603-2, Invitrogen, CA, Carlsbad, USA; LCN2, MLCN20, R&D Systems, Minneapolis, MN, USA) following the manufacturer’s protocol. Protein levels were estimated based on absorbance measured at 450 nm (pg/mL) using a microplate reader.

### 2.9. Statistical Analysis

All data are expressed as the mean ± SEM. Data were analyzed using one-way analysis of variance (ANOVA) followed by Bonferroni post-hoc tests for behavioral and biochemical studies. Data were analyzed using the R version 4.1.2 software (R Core Team, 2022). Statistical significance was set at *p* < 0.05.

## 3. Results

### 3.1. Identification of HME by Detecting Skimmin and Isopimpinellin in UHPLC Chromatograms

**Figure 2:**
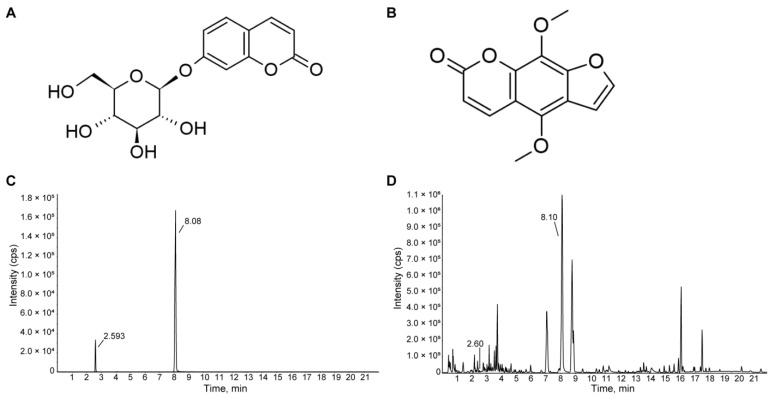
Chemical structure of skimmin (**A**) and isopimpinellin (**B**). Results of base peak chromatogram (BPC) of skimmin and isopimpinellin from (**C**) reference standards and (**D**) the 30% ethanol extract of HME. A peak at 2.60 and 8.10 is expected to correspond to skimmin and isopimpinellin.

The HME extract was identified by the detection of skimmin and isopimpinellin, which are the main components of HME. Chromatographic analysis was performed using the UHPLC system. As shown in Figure 2, skimmin and isopimpinellin were detected in the HME powder, peaking at 2.60 min and 8.10 min, respectively.

### 3.2. Effects of HME on Depression-like and Anxiety-like Behaviors in an L-AAA-Infused Mouse Model of Depression

**Figure 3:**
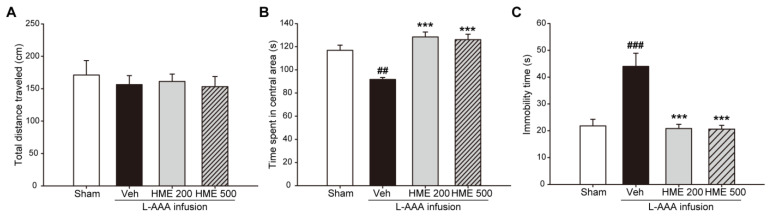
Effect of *Heracleum moellendorfii* extract (HME) treatment in (**A**,**B**) the open field test (OFT) and (**C**) tail suspension test (TST) in the L-AAA-infused mouse model of depression. (**A**) Total distance traveled by mice across the entire grid during OFT was used to measure locomotor activity, and no notable difference was found between the groups. (**B**) In the OFT, the distance that mice traveled in the central area was used to measure anxiety-like activity. Oral administration of 500 mg/kg HME increased the distance diminished by L-AAA infusion. (**C**) Depressive-like activity was determined by the immobility time in the TST. HME treatment reduced the immobility time, with a dose of 500 mg/kg showing statistical significance. The values are presented as mean ± SEM. n = 6/group. ## *p* < 0.01, ### *p* < 0.001 versus the sham, *** *p* < 0.001 versus L-AAA+Veh. HME, *Heracleum moellendorfii* extract; L-AAA, L-alpha-aminoadipic acid; Veh, vehicle.

To confirm that stereotaxic surgery or drug administration caused unexpected changes in ambulation, the locomotor activity of mice in each experimental group was evaluated by quantifying the total distance traveled freely during the 10-minute OFT. No significant differences were observed between the groups (*p* > 0.05, Figure 3A). Accordingly, sensorimotor deficits are less likely to be involved in the behavioral test results.

We also analyzed anxiety-like behavior from the OFT results using the distance traveled in the central area of the apparatus. The L-AAA+Veh mice moved less distance in the central area for 10 min compared to the sham mice (*p* < 0.01). However, both the L-AAA+HME 200 mg/kg mice and L-AAA+HME 500 mg/kg mice showed a significantly increased movement and nearly recovered to the level observed in sham mice. (*p* < 0.001, respectively, Figure 3B).

The effects of HME on depression-like behavior in L-AAA-infused mice were assessed using TST. We discovered that the L-AAA-infused mice’s immobility time showed a significant increase compared to the L-AAA+Veh group (*p* < 0.001). HME treatment diminished the immobility time with statistical significance in both the low- and high-dose groups (*p* < 0.001 in both L-AAA+HME 200 mg/kg and L-AAA+HME 500 mg/kg, Figure 3C).

### 3.3. Effects of HME on NeuN and GFAP Expression in the PFC of an L-AAA-Infused Mouse Model of Depression

To verify the proper establishment of the animal model, H&E staining was performed on sections from the insertion site in the PFC. In H&E staining, necrosis or other unexpected damage due to surgery was not observed across the groups (Supplementary Figure S1C; original magnification 200×). Thus, any subsequent changes in behavior and tissue can be assumed to be derived from L-AAA infusion or drug administration.

**Figure 4:**
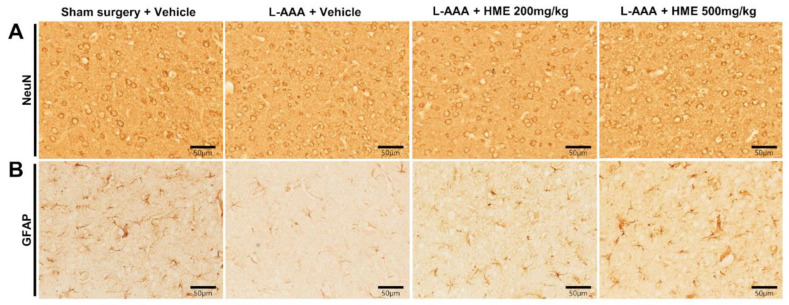
Effect of *Heracleum moellendorfii* extract (HME) treatment on NeuN and GFAP expression in the PFC of L-AAA-infused mouse model of depression determined via immunohistochemistry. (**A**) Effect of L-AAA injection on NeuN expression in the PFC. There was no significant change between groups after the L-AAA injection. (**B**) Effect of HME on GFAP expression in the PFC. GFAP expression decreased extremely after L-AAA injection; however, the administration of HME dose-dependently increased the expression. Representative results from immunohistochemistry are presented. Calibration bars are set at 50 μm. GFAP, glial fibrillary acidic protein; HME, *Heracleum moellendorfii* extract; L-AAA, L-alpha-aminoadipic acid; PFC, prefrontal cortex; NeuN, neuronal-specific nuclear protein.

In immunohistochemistry, we first stained NeuN to check for neuronal loss in the PFC. No significant change was observed in NeuN expression across all groups (Figure 4A; original magnification 400×). Accordingly, injection of L-AAA, the glial toxin, did not influence neurons in the PFC at any time point, which resembles the brain of patients with depression.

GFAP immunoreactivity was obviously reduced in L-AAA+Veh mice compared to sham mice. In contrast to L-AAA infusion resulting in severe astrogliosis, both doses of 200 mg/kg and 500 mg/kg HME increased GFAP expression in a dose-dependent manner. L-AAA+HME 500 mg/kg reversed GFAP expression to levels similar to those in sham mice (Figure 4B; original magnification 400×).

### 3.4. Anti-Inflammatory Effects of HME on Proinflammatory Cytokines and LCN2 in the PFC of an L-AAA-Infused Mouse Model of Depression

**Figure 5:**
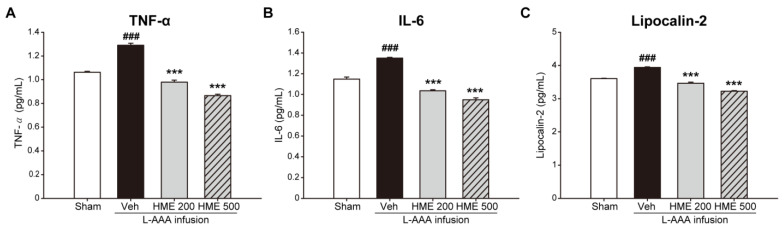
Anti-inflammatory effect of *Heracleum moellendorfii* extract (HME) treatment on the level of proinflammatory cytokines and lipocalin-2 (LCN2) in the PFC of L-AAA-infused mouse model of depression examined through ELISA. (**A**,**B**) Effect of HME on TNF-α and IL-6 levels in the PFC. L-AAA infusion increased both TNF-α and IL-6 levels, and oral administration of HME reversed these proinflammatory cytokines dose-dependently. (**C**) Effect of HME on LCN2 level in the PFC. LCN2 levels increased after L-AAA was introduced into the PFC, but oral administration of HME reduced LCN2 levels dose-dependently. The values are the mean ± SEM. n = 4/group. ### *p* < 0.001 versus the sham, *** *p* < 0.001 versus L-AAA+Veh. ELISA, Enzyme-Linked Immunosorbent Assay; HME, *Heracleum moellendorfii* extract; IL-6, interleukin 6; L-AAA, L-alpha-aminoadipic acid; LCN2, lipocalin-2; PFC, prefrontal cortex; TNF-α, tumor necrosis factor-α; Veh, vehicle.

As astrocytes are largely involved in neuroinflammation, changes in proinflammatory cytokines were determined. Proinflammatory cytokines, TNF-α and IL-6, were evaluated using ELISA. The protein level of TNF-α significantly increased after L-AAA infusion (*p* < 0.001 vs. sham). Conversely, treatment with HME significantly decreased TNF-α levels in a dose-dependent manner (both *p* < 0.001 vs. L-AAA+Veh, Figure 5A). L-AAA+Veh mice also had significantly higher IL-6 levels than sham mice. (*p* < 0.001 vs. sham). Treatment with HME resulted in a dose-dependent reduction of IL-6 levels (both *p* < 0.001 vs. L-AAA+Veh, Figure 5B).

As LCN2 is suggested to be a master proinflammatory mediator, its protein levels were also examined. In L-AAA+Veh mice, the level of LCN2 increased compared to sham mice (*p* < 0.001 vs. sham). HME-treated mice showed ameliorated LCN2 levels in a dose-dependent manner (both *p* < 0.001 vs. L-AAA+Veh). The change in LCN2 following HME treatment was concurrent with that of TNF-α and IL-6 (Figure 5C).

## 4. Discussion

The purpose of this study was to examine the antidepressant effects of HME by reversing the pathological changes in astrocytes and investigating its anti-inflammatory mechanism. Numerous postmortem human brain studies have demonstrated predominant changes in the density, morphology, and function of astrocytes in patients with mood disorders [20]. To replicate this pathological phenomenon in an animal model, we injected the glial toxin L-AAA into the PFC, where astrocyte loss is apparent in patients with depression. When L-AAA-injected mice were treated with HME, their immobility time was diminished in the TST, indicating that HME ameliorates depression-like behavior. HME treatment also increased the distance traveled in the central area of the OFT, implying decreased anxiety-like behavior. Concurrent with these behavioral changes, HME promotes recovery from astrogliosis and neuroinflammation, which can be a major cause of depression.

Previous studies have shown that patients with depression exhibit reduced numbers of astrocytes in the PFC and hippocampus [5]; therefore, an astrocyte ablation model using L-AAA infusion was adopted in this study. Since L-AAA eliminates astrocytes through transitory ablation, intracerebral injection was used as a novel model of depression [21,22]. L-AAA enters cells via sodium-dependent transporters and triggers glial cell death by disrupting vital cellular functions, such as glutamate regulation [23,24]. Several studies have investigated the antidepressant effects of materials using this animal model [3,25].

HME restored the density of GFAP, which was decreased by L-AAA infusion into the PFC. Astrocytes, which comprise the largest portion of glial cells, play many crucial roles in the central nervous system. They maintain the homeostasis of neurotransmitters, support synaptic transmission and neurogenesis, and regulate vascular tone [26,27]. Thus, in pathological states, they affect neural circuits through synaptic dysfunction and interaction with neurons, as well as changes in the morphology and function of astrocytes [26]. The importance of rescuing astrocytes in the treatment of depression is supported by postmortem and animal research [20,28]. A recent study reported that restoration of astrocytes following L-AAA injection in the PFC reversed depression-like behaviors. Conversely, the re-ablation of repopulated astrocytes led to the recurrence of depression [29]. Therefore, enhancing astrocyte repopulation with HME could represent a promising therapeutic approach for depression, as it restores the astrocyte population and ameliorates associated depressive behaviors.

Proinflammatory cytokines like TNF-α and IL-6 were dose-dependently decreased by the administration of HME, while L-AAA increased neuroinflammation in the PFC, as shown by an increase in proinflammatory cytokines. Immune activation and altered proinflammatory responses have been reported to be related to major depressive disorder [30]. Considering previous studies, reducing neuroinflammation may be an important feature of antidepressant treatments. Increased IL-6 and TNF-α have been reported in depressive patients and are positively correlated with Hamilton Depression Scale-17 [31]. In the pooled data from the meta-analysis, fluoxetine decreased IL-6, TNF-α, and IL-1β levels [32]. IL-6 knockout mice and TNF-α receptor knockout mice have shown resistance to the induction of depression [33,34,35]. Astrocyte ablation by L-AAA resulted in alterations in behavioral tests (such as novel object recognition test, forced swimming test, and TST) and the occurrence of ameboid microglia, accompanied by increased TNF-α, IL-6, and IL-10 levels, suggesting that astrocytes are an important element in depression-related neuroinflammation [36]. Thus, HME presumably acts on the neuroinflammatory component by reversing astrocytic function.

In addition, LCN2, the master mediator of inflammation regulated by astrocytes, was decreased in the same manner. Previous studies have shown that LCN2 is a mediator of inflammation, recruiting inflammatory cells and inducing proinflammatory cytokines [37]. LCN2 was examined to assess the antidepressant effect of HME. It has been demonstrated that LCN2 can stimulate the phosphorylation of NF-kB by binding to cell membrane receptors, 24p3R, which in turn initiates the expression of pro-inflammatory genes, including IL-6, TNF-α, and IL-1β [38,39]. In addition, LCN2 has been suggested as a new biomarker for the current status of depression, as its plasma levels correlate with the depression severity in elderly patients with major depressive disorder and are unaffected by the use of antidepressants or onset of depression, unlike other inflammatory markers (e.g., C-reactive protein and interferon alpha) [40]. Behavioral changes in ovariectomized rats have also been associated with increased levels of LCN2 [41]. Controlling LCN2, associated with astrocytes, might offer a potential solution for treating depression, as current LCN2-targeted treatment methods include targeted drug delivery to mitigate its overexpression and interaction with astrocytes, thereby potentially reducing neuroinflammation and enhancing neurological recovery [39]. According to these behavioral and histological results, HME supposedly has antidepressant and anxiolytic effects by reversing astrocyte loss and anti-inflammatory effects through the engagement of LCN2.

HME is known to have detoxification and anti-oxidative effects [11,12]. HME has traditionally been used for inflammatory diseases, including arthritis, back pain, and fever [10]. It has been reported to inhibit proinflammatory cytokines by blocking NF-κB and MAPK signaling and stimulating ROS/Nrf2/HO-1 signaling [42]. Skimmin, one of the major ingredients present in HME, is known for its antibilious, antiparasitic, and antipyretic effects [43]. A previous study has investigated its anti-inflammatory effects in a rat model [44]. Isopimpinellin, another major ingredient of HME, has antioxidant and anti-inflammatory properties [45,46]. Furthermore, these findings imply that they can be applied to other brain diseases susceptible to neuroinflammation and astrocyte loss. In future studies, it is necessary to determine which ingredients of HME act as the antidepressant through anti-inflammatory mechanisms.

One of the limitations of this study is that among the various causes of depressive disorder, only astrocyte-related mechanisms were mainly studied. Although astrocytes contribute significantly to depression, the approach used to the effects of HME reported in this study may provide limited information, as there are diverse suggested mechanisms for the onset of depression. Second, as many previous studies have reported that proinflammatory cytokines, including TNF-α, IL-6, and IL-1β, were provoked after LCN2 was activated, we assumed a causal relationship between LCN2 and proinflammatory cytokines [37]. However, to confirm this, it is necessary to verify that the change is induced by controlling LCN2. Third, quantifying the amounts of marker compounds in our extract would provide a more comprehensive characterization. Our study utilized qualitative analysis, which conforms to the existing guideline for lesser-studied botanical extracts but may lack the precise quantification needed for replication and detailed comparison with other studies [47]. Fourth, another limitation is the use of relatively high doses of extracts. Future research should investigate the effects of lower doses to better understand their practical implications. Fifth, as the positive control group treated with typical antidepressants was not included, the effect of HME was restrictedly evaluated. However, many behavioral properties and biological markers recovered after HME treatment, which was equivalent to those of sham mice. If the results are compared with typical antidepressants like SSRIs or TCAs, a similar result is expected.

## 5. Conclusions

In this study, we evaluated the antidepressant effects of HME through an anti-inflammatory mechanism using an astrocyte-ablated mouse model. HME improved depression and anxiety behaviors in L-AAA-infused mice. These results suggest that HME intervenes in neuroinflammation by reversing astrocytic loss in a depressive-like state in mice. In conclusion, HME may cure inflammatory diseases related to astrocytes, such as depression.

## Data Availability

The datasets used and/or analysed during the current study are available from the corresponding author upon reasonable request.

## Supplementary Materials

The following supporting information can be downloaded at: https://www.mdpi.com/article/10.3390/nu16132049/s1, Figure S1: Cannula production and localization at the injection site.
