# Supplementary material for: Antidepressant Effect of Heracleum moellendorffii Extract on Behavioral Changes in Astrocyte Ablation Mouse Model of Depression by Modulating Neuroinflammation through the Inhibition of Lipocalin-2
Source: Nutrients. 2024 Jun 27;16(13):2049. doi: 10.3390/nu16132049 (PMC11243176; doi:10.3390/nu16132049)
Supplement: Supplementary file 1 [file nutrients-16-02049-s001.zip › nutrients-3028186-supplementary.pdf]

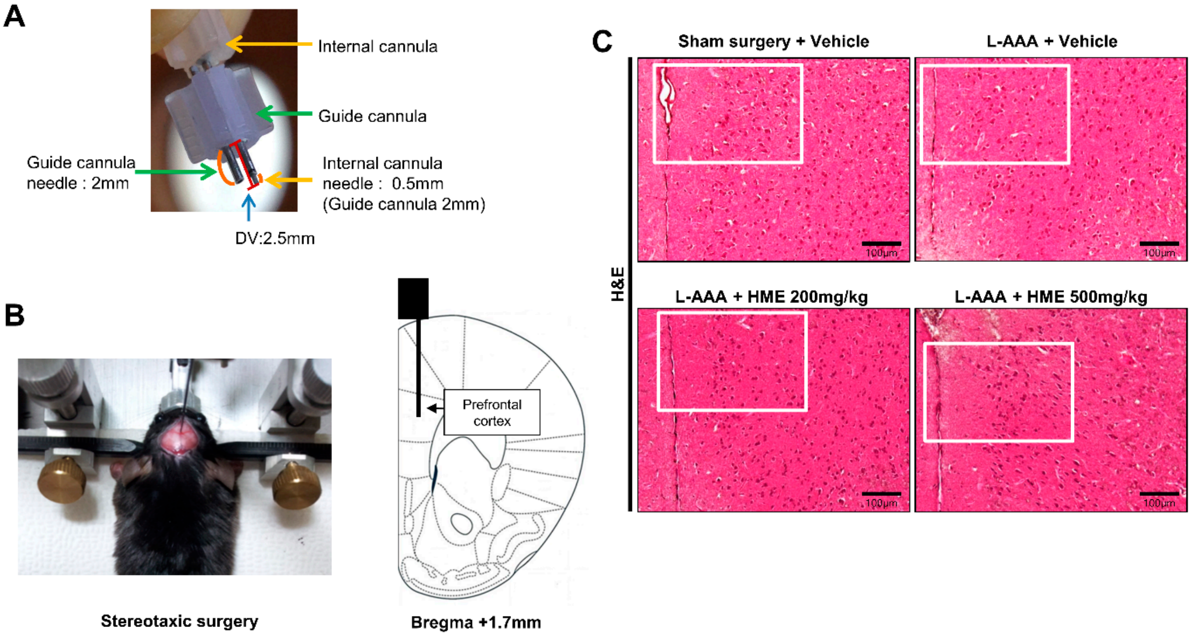

Figure S1. Cannula production and localization at the injection site. (A) Schematic design of the double cannula. The apparatus consists of a guide cannula, internal cannula, and injection cannula, which are designed to inject L-alpha-aminoadipic acid (L-AAA) into the prefrontal cortex. (B) The cannula was implanted into the prefrontal cortex region using the following coordinates: 1.7 mm anterior/posterior, ±0.3 mm medial/lateral and -2.5 mm dorsal/ventral from bregma. (C) Hematoxylin and eosin (H&E) staining was performed to determine the proper location of the injection sites. There was no evidence of necrosis. Representative results of H&E staining are shown. Scale bar = 100 μm. HME, *Heracleum moellendorfii* extract; L-AAA, L-alpha-aminoadipic acid.
